# Human Histatin 5 Exerts Anti-Trypanosomal Activity Against *Trypanosoma cruzi* and Induces Ultrastructural Damage, Apoptosis-like Cell Death, and Oxidative/Nitrosative Stress

**DOI:** 10.3390/ijms27167361

**Published:** 2026-08-18

**Authors:** Blanca Esther Blancas-Luciano, Ingeborg Becker, Marco Antonio Sánchez-Chávez, Aketzalli Gómez-Guzmán, Reyna Lara-Martínez, Luis Felipe Jiménez-García, Jaime Zamora-Chimal, José Delgado-Dominguez, Ana María Fernández-Presas

**Affiliations:** 1 Departamento de Microbiología y Parasitología, Facultad de Medicina, Universidad Nacional Autónoma de México, Ciudad de México 04510, Mexico; bblancas@facmed.unam.mx (B.E.B.-L.); marcosanchezchavez@ciencias.unam.mx (M.A.S.-C.); agg-1@ciencias.unam.mx (A.G.-G.); 2 Unidad de Medicina Experimental, Universidad Nacional Autónoma de México, Hospital General de México “Dr. Eduardo Liceaga”, Ciudad de México 06726, Mexico; becker@unam.mx (I.B.); zamorachimaljaime@ciencias.unam.mx (J.Z.-C.); josesoterod@gmail.com (J.D.-D.); 3 Departamento de Biología Celular y Tisular, Facultad de Ciencias, Universidad Nacional Autónoma de México, Ciudad de México 04510, Mexico; rlm@ciencias.unam.mx (R.L.-M.); luisfelipe_jimenez@ciencias.unam.mx (L.F.J.-G.)

**Keywords:** *Trypanosoma cruzi*, Histatin 5, epimastigotes

## Abstract

Chagas disease, caused by *Trypanosoma cruzi*, remains a neglected tropical disease of relevance due to its chronic complications and limited therapeutic options. Current treatments, mainly benznidazole and nifurtimox, are limited by toxicity, adverse effects, and reduced efficacy in chronic infection. Therefore, antimicrobial peptides (AMPs) have emerged as promising candidates for identifying new antiparasitic strategies because of their ability to affect multiple cellular targets. In this study, we evaluated the anti-trypanosomal activity, host-cell cytotoxicity, and cellular alterations induced by the human salivary peptide Histatin 5 (Hist 5) against *T. cruzi* epimastigotes. The reversibility of the antiparasitic effect was assessed through washout assays after 48 h of exposure. Cytotoxicity was evaluated in Vero cells and RAW264.7 macrophages, whereas parasite susceptibility was determined using dose–response curves. The reversibility of the antiparasitic effect was assessed through washout assays after 48 h of exposure. Ultrastructural alterations induced by Hist 5 were analyzed by transmission electron microscopy. Cell death-associated events were assessed using Annexin V/PI staining and TUNEL assays to detect phosphatidylserine externalization and DNA fragmentation, respectively. Reactive oxygen species and nitric oxide production were quantified using *2′,7′-dichlorodihydrofluorescein diacetate* (H_2_DCFDA) fluorescence and the Griess reaction. Hist 5 showed limited cytotoxicity toward mammalian cells and reduced parasite viability in a time- and concentration-dependent manner. Following peptide removal, parasite growth recovered only partially, indicating that the antiparasitic effect was not completely reversible. Hist 5 also induced marked ultrastructural damage, apoptosis-like cell death, increased intracellular ROS levels, and enhanced NO production. These findings suggest that Hist 5 affects *T. cruzi* epimastigotes through multiple cellular alterations while exerting limited effects on host-cell viability.

## 1. Introduction

Chagas disease (CD), also known as American trypanosomiasis, is a neglected tropical disease (NTD) caused by the protozoan parasite *Trypanosoma cruzi* (Kinetoplastida: Trypanosomatidae), which is primarily transmitted by insect vectors. It affects approximately 6–8 million people worldwide, while an estimated 65–100 million individuals live in areas at risk of infection [1,2]. The World Health Organization (WHO) recognizes CD as one of the most important NTDs globally, and it remains a major public health concern in endemic regions of Mexico, Central America, and South America [3]. Although many infected individuals remain asymptomatic for years, approximately 30% eventually develop chronic, debilitating, and potentially life-threatening complications involving the heart, colon, or esophagus [4].

Currently, only two drugs are available for the etiological treatment of Chagas disease: benznidazole and nifurtimox. Both are mainly effective during the acute and early chronic phases of infection [5]. However, neither drug meets the WHO criteria for an optimal therapeutic agent, which include: (i) parasitological cure in both acute and chronic stages, (ii) high efficacy with a single or limited number of doses, and (iii) absence of teratogenicity or severe adverse effects [6]. Although benznidazole has shown a cure rate of up to 75% after 27 years of follow-up, its clinical use remains limited by high toxicity, frequent adverse reactions, treatment discontinuation, and reduced susceptibility during chronic infection. Indeed, adverse effects associated with benznidazole have been reported in 48% to 86% of treated patients, often leading to interruption of therapy. Comparative studies conducted in Latin America have also reported wide variations in treatment completion rates, ranging from 58.6% to 88.4% for benznidazole and from 25% to 96.3% for nifurtimox [7,8]. Consequently, these limitations underscore an urgent and critical need to identify novel synthetic or natural compounds that offer favorable susceptibility profiles, low toxicity, and affordable production and administration costs.

In recent years, numerous AMPs have been identified and characterized as promising therapeutic candidates against infections caused by microorganisms with limited or no effective treatment options [9]. AMPs are generally amphipathic and highly cationic molecules that can selectively interact with microbial membranes based on their charge, lipid composition, and structural organization. These physicochemical properties contribute to their broad-spectrum antimicrobial and antiparasitic activities, mainly through electrostatic interactions with negatively charged microbial membranes. AMPs can exert their microbicidal effects by disrupting lipid bilayer organization, thereby compromising membrane integrity, promoting permeabilization, and contributing to microbial cell death [10,11,11]. In addition to membrane-disruptive mechanisms, some AMPs can interfere with intracellular metabolic pathways or target specific cytoplasmic components [12]. Beyond their direct microbicidal effects, AMPs may also modulate host immune responses by influencing cytokine production and other defense mechanisms [13].

Due to their multifunctional properties, AMPs—also referred to as host-defense peptides—have attracted increasing attention in antiparasitic research [14]. Among them, histatins represent a family of histidine-rich AMPs originally isolated from human parotid gland secretions [15], which display broad-spectrum antibacterial and antifungal activities [16]. In particular, the human salivary peptide Hist 5 has shown potent activity against *Leishmania* spp., including both promastigote and amastigote stages. Its leishmanicidal effect has been associated with mitochondrial accumulation, disruption of ATP synthesis, and bioenergetic collapse, supporting its relevance as a model peptide for antiparasitic studies [17].

Previous studies have shown that AMPs from diverse structural families can exert potent effects against *T. cruzi* and other *Trypanosoma* species. Peptides such as apidaecin, magainin II, melittin, and cecropin A have demonstrated robust in vitro anti-trypanosomal properties [18], while other families—including dermaseptins, phylloseptins, temporins, cruzioseptins, and pictuseptins—have expanded the repertoire of peptide-based candidates capable of targeting kinetoplastid parasites [19,20,21]. These findings provide a relevant precedent for the continued exploration of AMPs as sources of antiparasitic molecules and as tools to uncover parasite vulnerabilities.

Therefore, this study aimed to evaluate the anti-trypanosomal activity of Hist 5 against *T. cruzi* epimastigotes by examining its effects on parasite proliferation and viability, the reversibility of its activity, the mechanism of cell death induced, and the modulation of reactive oxygen and nitrogen species production. In addition, the cytotoxic effects of Hist 5 were assessed in Vero cells and RAW264.7 macrophages.

## 2. Results

### 2.1. Hist 5 Inhibits T. cruzi Epimastigote Growth, with Partial Recovery After Peptide Withdrawal

**Figure 1:**
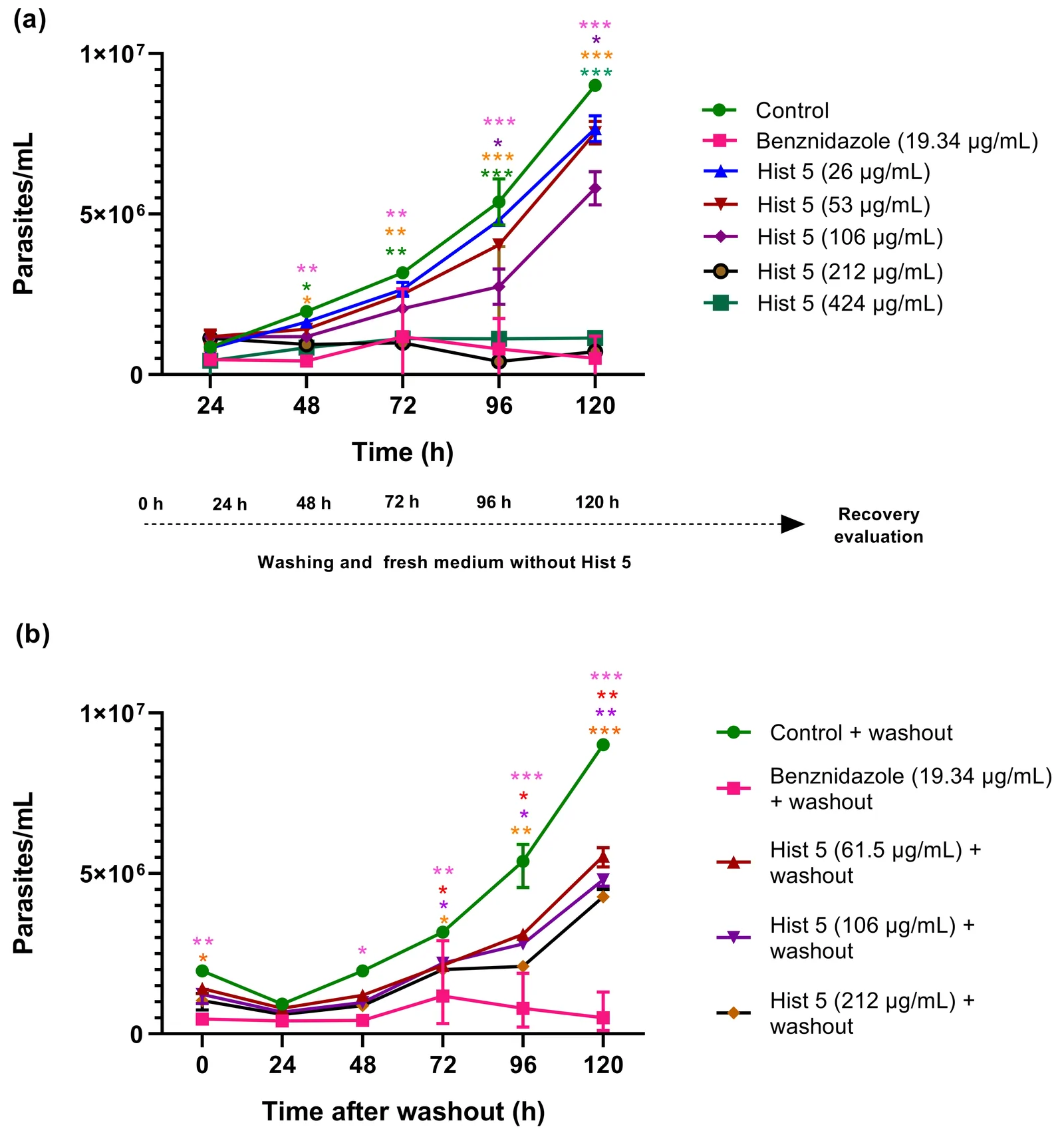
Effect of Histatin 5 on *Trypanosoma cruzi* epimastigote growth and recovery after treatment withdrawal. (**a**) *T. cruzi* epimastigotes were exposed to Hist 5 at 26, 53, 106, 212, and 424 µg/mL for up to 120 h. Benznidazole (19.34 µg/mL) was used as the reference drug, and untreated parasites were included as the growth control. Viable parasite density was determined at the indicated time points by direct counting in a Neubauer chamber using motility and trypan blue exclusion as viability criteria. (**b**) For the washout assay, epimastigotes were exposed for 48 h to Hist 5 at 61.5 µg/mL (48-h IC_50_), 106 µg/mL, or 212 µg/mL, or to benznidazole (19.34 µg/mL). After treatment, parasites were washed twice with PBS and resuspended in fresh LIT medium without Hist 5 or benznidazole. Untreated parasites subjected to the same washout procedure were used as controls. Parasite growth and viability were monitored immediately after washout (0 h) and at 24, 48, 72, 96, and 120 h after treatment withdrawal. Data are expressed as the mean ± SD from five independent experiments. Asterisks indicate statistically significant differences relative to the corresponding control group at the same time point: * *p* < 0.05, ** *p* < 0.01, and *** *p* < 0.001.

The in vitro anti-trypanosomal activity of Hist 5 against *T. cruzi* epimastigotes was evaluated over a 120 h period by determining the concentration-dependent reduction in counted viable parasite numbers relative to untreated controls (Figure 1a). Hist 5 at 212 and 424 μg/mL significantly reduced the viable parasite numbers from 48 to 120 h compared with the untreated controls, with significance levels ranging from *p* < 0.05 to *p* < 0.001, as indicated in Figure 1a. At 106 μg/mL, Hist 5 produced a partial but significant reduction in the number of viable epimastigotes at 96 and 120 h, whereas the lower concentrations of 26 and 53 μg/mL showed growth patterns comparable to those of untreated parasites (*p* > 0.05). Benznidazole, used as the reference drug, significantly reduced the viable parasite numbers at 72, 96, and 120 h (*p* < 0.001).

The IC_50_ values calculated from the dose–response curves (Figure 1a) are summarized in Table 1. Hist 5 exhibited IC_50_ values of 98.89 ± 0.24 μg/mL at 24 h and 61.5 ± 0.5 μg/mL at 48 h. The IC_50_ subsequently increased to 135.23 ± 0.56 μg/mL at 72 h and 156.21 ± 0.32 μg/mL at 96 h, indicating a reduction in the inhibitory activity of Hist 5 during prolonged exposure. In contrast, benznidazole demonstrated higher potency against *T. cruzi* epimastigotes, displaying consistently lower IC_50_ values at all time points (9.45 ± 0.12 μg/mL at 24 h, 19.34 ± 0.5 μg/mL at 48 h, 15.34 ± 0.23 μg/mL at 72 h, and 6.57 ± 0.54 μg/mL at 96 h).

To determine whether the inhibitory effect of Hist 5 persisted after peptide removal, epimastigotes exposed for 48 h to Hist 5 at 61.5, 106, or 212 µg/mL were washed, resuspended in fresh peptide-free LIT medium, and monitored for an additional 120 h (Figure 1b). At the time of washout (0 h), parasite densities were numerically lower in all treated cultures than in the washed control, reflecting the effect of the preceding 48 h exposure period. These differences were significant in cultures treated with benznidazole (*p* < 0.01) and Hist 5 at 212 µg/mL (*p* < 0.05).

During the first 24 h after washout, parasite densities decreased in all groups, with no significant differences relative to the washed control (*p* > 0.05). At 48 h, only benznidazole-treated cultures remained significantly below the washed control (*p* < 0.05). From 72 h onward, proliferation resumed in all Hist 5-treated cultures; however, parasite densities remained significantly lower than those of the washed control at all three concentrations. The corresponding levels of significance ranged from *p* < 0.05 to *p* < 0.001, as indicated in Figure 1b. At 120 h, the reduction in parasite density became greater with increasing Hist 5 concentration. Benznidazole-treated cultures showed limited recovery and remained significantly below the washed control from 48 to 120 h. Overall, these findings indicate that parasite proliferation partially recovered after Hist 5 withdrawal but did not return to the control levels within the evaluated period.

### 2.2. Hist 5 Exerts Limited Cytotoxicity Toward Mammalian Cells

**Figure 2:**
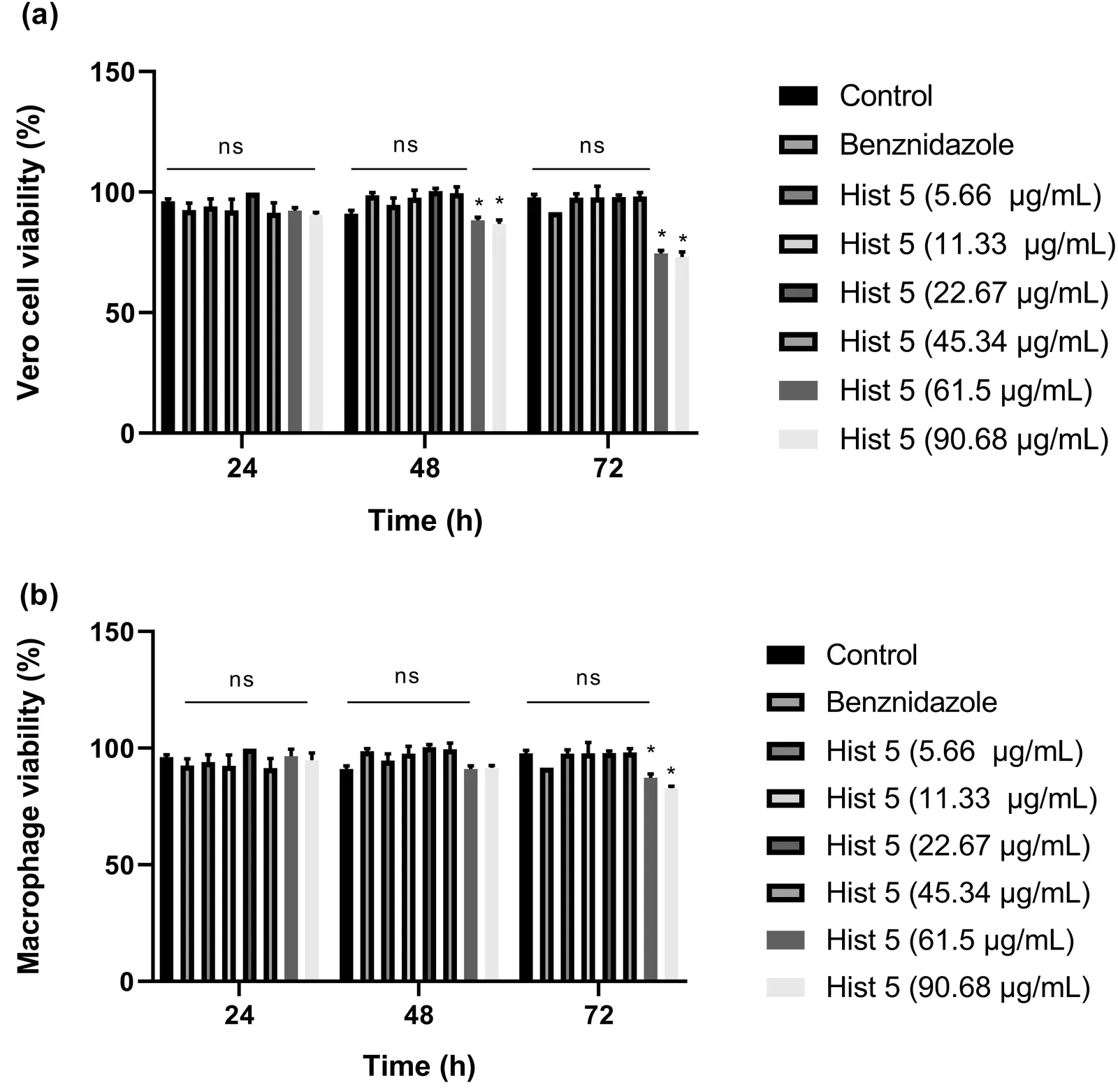
Effect of Hist 5 on mammalian cell viability. (**a**) Viability of Vero cells exposed to different concentrations of Hist 5. (**b**) Viability of RAW264.7 macrophages exposed to different concentrations of Hist 5. Cells were incubated at 37 °C in a 5% CO_2_ atmosphere. Data are expressed as mean ± SD from five independent experiments. Asterisks indicate statistically significant differences compared with untreated cells only when shown in the figure (* *p* < 0.05). ns, not significant.

To determine the in vitro cytotoxicity of Hist 5, cell viability was evaluated in Vero cells and RAW264.7 macrophages. Hist 5 did not significantly reduce cell viability in either mammalian cell line at the concentrations evaluated compared with the untreated controls (*p* > 0.05; Figure 2a,b). Moreover, cell viability did not decrease by 50% within the tested concentration range; therefore, CC_50_ values could not be determined under these experimental conditions. These results indicate that Hist 5 exerts limited cytotoxic effects on mammalian cells at the concentrations evaluated.

### 2.3. Ultrastructural Alterations in T. cruzi Epimastigotes Treated with Hist 5

**Figure 3:**
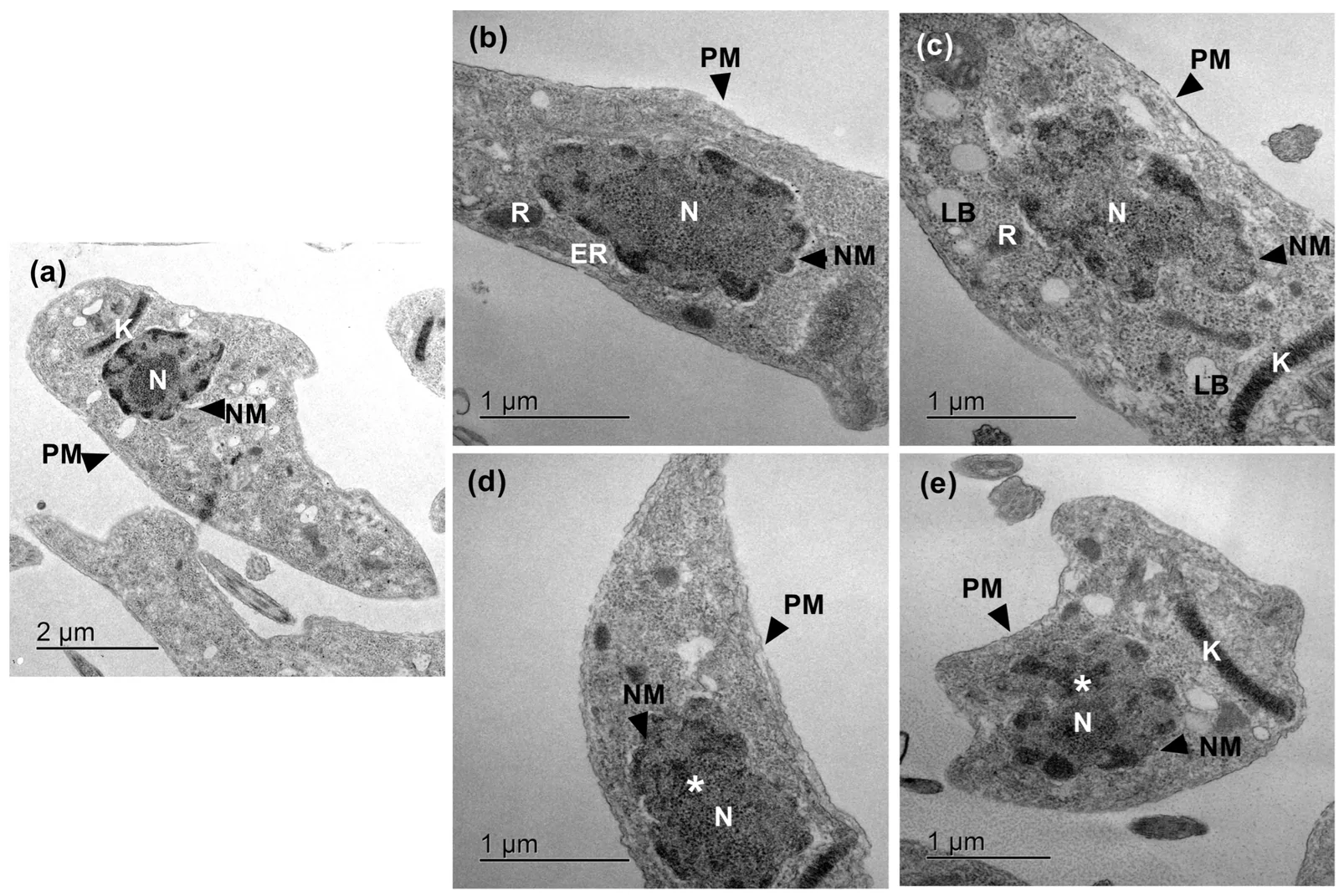
Ultrastructural alterations in *T. cruzi* epimastigotes treated with Hist 5. Transmission electron micrographs of *T. cruzi* epimastigotes incubated at 28 °C. (**a**) Untreated epimastigotes incubated in RPMI 1640 for 72 h showed preserved ultrastructural organization, including an intact nucleus (N), nuclear membrane (NM), kinetoplast (K), and plasma membrane (PM). (**b**,**c**) Epimastigotes treated with Hist 5 (61.5 µg/mL) for 48 h showed plasma membrane discontinuities, alterations in the nuclear membrane, abnormal chromatin organization, swollen endoplasmic reticulum (ER), swollen reservosomes (R), and kinetoplast alterations. Lipid bodies (LB) were also observed. (**d**,**e**) Epimastigotes treated with Hist 5 for 72 h showed more evident ultrastructural damage, including plasma membrane discontinuities, alterations in the nuclear and kinetoplast membranes, kinetoplast swelling, and abnormal nuclear chromatin organization (*). Scale bars: 1–2 µm.

Transmission electron microscopy (TEM) was used to evaluate the ultrastructural changes induced by Hist 5 in *T. cruzi* epimastigotes after 48 and 72 h of incubation. Untreated epimastigotes exhibited a typical elongated morphology, characterized by a well-defined and intact plasma membrane, organized subpellicular microtubules, and preserved architecture of the mitochondrion, kinetoplast, nucleus, and flagellum (Figure 3a).

Following 48 h of exposure to Hist 5, the parasites displayed marked ultrastructural abnormalities. These early alterations included reduced nuclear electron density, prominent chromatin margination and condensation, swollen reservosomes, and distension of both mitochondrial and kinetoplast membranes. Additionally, initial signs of plasma membrane compromise, such as infoldings and focal discontinuities, were observed (Figure 3b,c).

These morphological defects progressed severely after 72 h of treatment. Epimastigotes exhibited a pronounced loss of cytoplasmic architecture, evidenced by extensive vacuolization and electron-lucid spaces, suggesting the depletion or extrusion of intracellular components. The nuclear and kinetoplast membranes showed distinct breaks, accompanied by a granular nuclear matrix and severe kinetoplast swelling. Furthermore, while swelling of the endoplasmic reticulum was apparent in some parasites, in others, this organelle was completely disorganized and indistinguishable. Persistent plasma membrane invaginations and extensive disruptions further confirmed the irreversible structural collapse induced by Hist 5 (Figure 3d,e).

Taken together, these ultrastructural findings demonstrate that Hist 5 exerts a multi-target disruptive effect on *T. cruzi* epimastigotes, profoundly compromising nuclear organization, mitochondrial–kinetoplast integrity, endomembrane systems, and plasma membrane continuity.

### 2.4. Hist 5 Induces DNA Fragmentation and Annexin V Positivity in T. cruzi Epimastigotes

**Figure 4:**
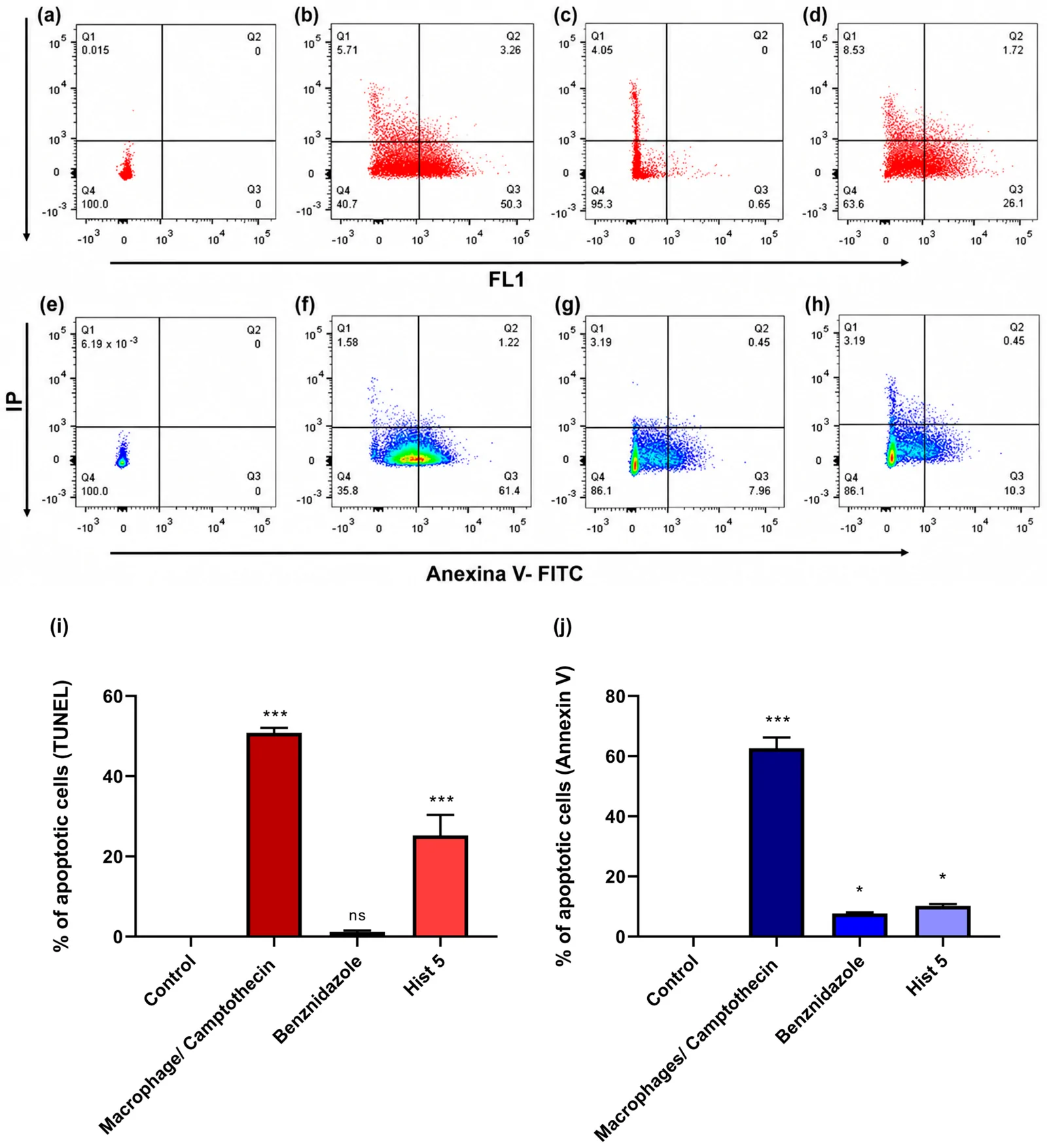
Evaluation of Hist 5-induced apoptosis-like cell death in *T. cruzi* epimastigotes. Representative dot plots of the TUNEL assay after 48 h of incubation: (**a**) *T. cruzi* epimastigotes incubated with LIT culture medium alone, at 28 °C, (**b**) macrophages treated with camptothecin (4 μg/mL), used as a positive control for apoptosis, (**c**) *T. cruzi* epimastigotes treated with benznidazole, and (**d**) *T. cruzi* epimastigotes treated with Hist 5 (61.5 µg/mL). Representative dot plots of the Annexin V-FITC/PI assay after 48 h of incubation: (**e**) *T. cruzi* epimastigotes incubated with LIT culture medium alone, (**f**) macrophages treated with camptothecin (4 μg/mL), (**g**) epimastigotes treated with benznidazole, and (**h**) epimastigotes treated with Hist 5. Cell distribution was interpreted as follows: Annexin V−/PI−, viable cells; Annexin V+/PI−, cells undergoing early apoptosis-like cell death; Annexin V+/PI+ or Annexin V−/PI+, non-viable cells compatible with late apoptosis or necrosis. (**i**) Percentage of TUNEL-positive cells. (**j**) Percentage of Annexin V+/PI− cells. Data are expressed as the mean ± SD from five independent experiments. Asterisks indicate statistically significant differences relative to the untreated control group: * *p* < 0.05 and *** *p* < 0.001; ns, not significant.

The effect of Hist 5 on *T. cruzi* epimastigotes after 48 h of incubation was evaluated using TUNEL and Annexin V/PI assays. Untreated epimastigotes incubated with LIT medium were used as the negative control (Figure 4a,i), whereas camptothecin-treated macrophages were included as a positive control for apoptosis (Figure 4b,i). As shown in Figure 4i, benznidazole-treated parasites did not show a significant increase in TUNEL-positive cells compared with the untreated controls. In contrast, Hist 5 significantly increased the percentage of TUNEL-positive epimastigotes to 26.1% (*** *p* < 0.001), indicating DNA strand breaks associated with an apoptosis-like cell death process.

Flow cytometry analysis using Annexin V-FITC and PI further supported the induction of apoptosis-like changes. Untreated epimastigotes remained predominantly viable, with 100% of the population located in the Annexin V−/PI− quadrant. As expected, camptothecin-treated macrophages showed a marked increase in Annexin V+/PI− cells, reaching 61.4% (Figure 4f,j). Epimastigotes treated with benznidazole showed 7.96% Annexin V+/PI− cells (* *p* < 0.05). In comparison, Hist 5-treated epimastigotes showed 10.3% Annexin V+/PI− cells (* *p* < 0.05), suggesting the presence of an early apoptosis-like population with limited loss of membrane integrity.

Taken together, these results suggest that Hist 5 induces DNA fragmentation and promotes apoptosis-like alterations in *T. cruzi* epimastigotes after 48 h of exposure.

### 2.5. Histatin 5 Modulates ROS and Nitric Oxide-Associated Responses in T. cruzi Epimastigotes

**Figure 5:**
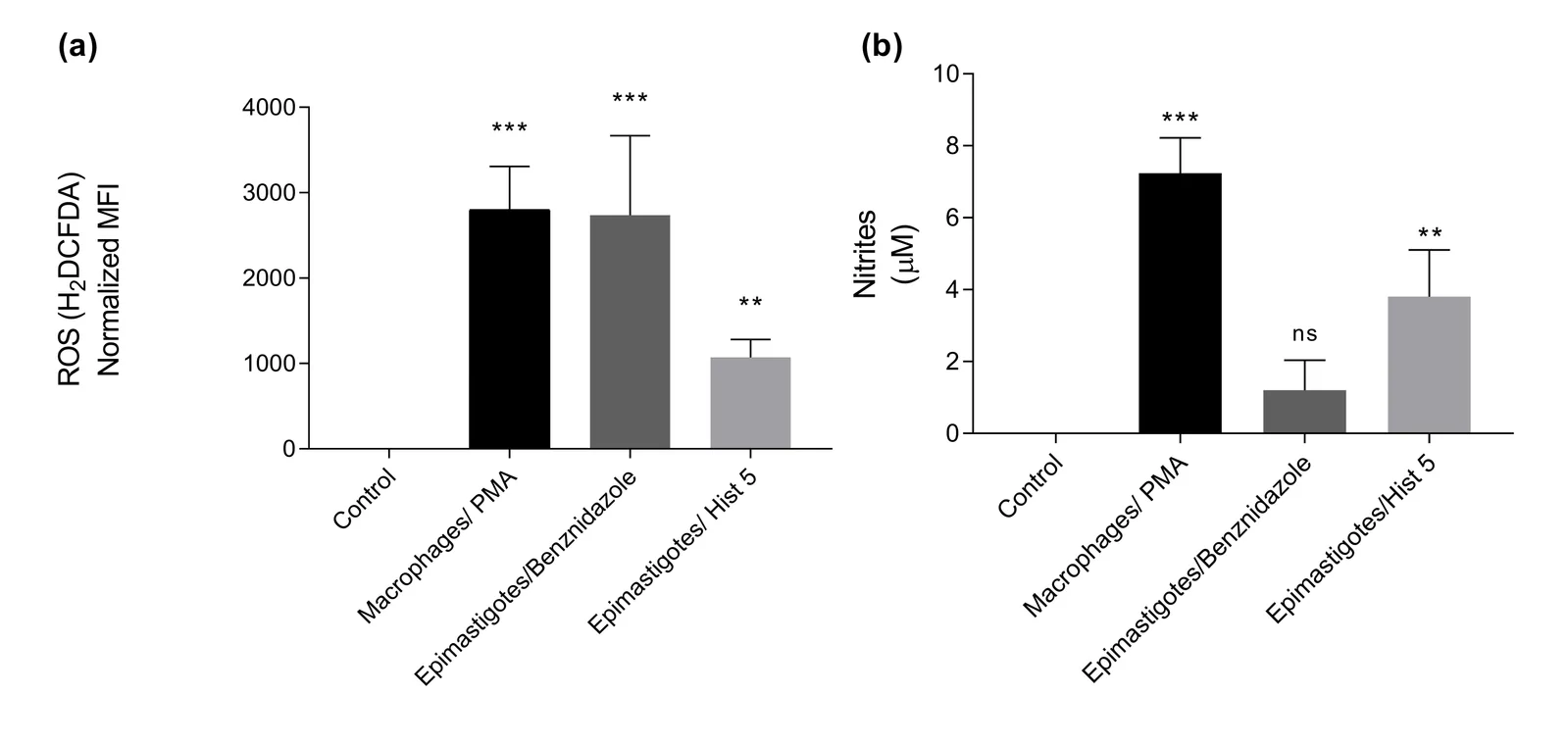
Effect of Hist 5 on ROS and nitrite levels in *T. cruzi* epimastigotes. (**a**) Reactive oxygen species (ROS) levels in epimastigotes were measured after 48 h using H_2_DCFDA and expressed as normalized mean fluorescence intensity (MFI). PMA-stimulated macrophages were included as a positive control for ROS production. Benznidazole-treated epimastigotes showed a marked increase in ROS levels, whereas Hist 5-treated epimastigotes showed a moderate but significant increase compared with the untreated controls. (**b**) Nitrite levels, used as an indirect indicator of nitric oxide production, were measured in culture supernatants. PMA-stimulated macrophages were used as a positive control for nitrite production. Benznidazole-treated epimastigotes showed a low, non-significant increase, whereas Hist 5-treated epimastigotes showed a significant increase in nitrite levels compared with the untreated controls. Data are expressed as mean ± SD from five independent experiments. The significance represents a *p* < 0.01 (**) and *p* < 0.001 (***) with respect to the control group.

ROS and nitric oxide-associated nitrite levels were evaluated after the incubation of *T. cruzi* epimastigotes with Hist 5. ROS levels were measured using H_2_DCFDA and expressed as normalized mean fluorescence intensity. As shown in Figure 5a, PMA-stimulated macrophages and benznidazole-treated epimastigotes showed a significant increase in ROS levels compared with the untreated control group (*** *p* < 0.001). Hist 5-treated epimastigotes also showed a significant increase in ROS levels compared with the untreated controls (** *p* < 0.01); however, this increase was lower than that observed in benznidazole-treated parasites.

Nitrite levels, used as an indirect indicator of nitric oxide production, were not detected in the untreated controls. Benznidazole-treated epimastigotes did not show a statistically significant difference in nitrite levels compared with the untreated control group (*p* > 0.05). As expected, PMA-stimulated macrophages, included as a positive control, showed a significant increase in nitrite production (*** *p* < 0.001). In comparison, Hist 5-treated epimastigotes showed a significant increase in nitrite levels compared with the untreated controls (** *p* < 0.01), suggesting that Hist 5 promotes nitric oxide-associated responses in *T. cruzi* epimastigotes.

Taken together, these results suggest that Hist 5 modulates redox-associated responses in *T. cruzi* epimastigotes, inducing moderate ROS production and increasing nitrite levels after 48 h of exposure.

## 3. Discussion

Our study demonstrates that *T. cruzi* epimastigotes are susceptible to the antimicrobial peptide Hist 5, supporting its potential as a molecule of interest for further antiparasitic investigation. Exposure to Hist 5 reduced parasite viability and was associated with ultrastructural alterations, apoptosis-like features, and changes in ROS and nitrite/NO-associated responses. In addition, the washout assay showed that parasite proliferation partially recovered after Hist 5 withdrawal, indicating that its inhibitory effect was not completely irreversible under the experimental conditions evaluated. Taken together, these findings suggest that Hist 5 affects *T. cruzi* epimastigotes through multiple cellular alterations and provide further evidence of its biological activity against this parasite. Although relatively high concentrations were required to achieve antiparasitic activity and partial reductions in mammalian cell viability/metabolic activity were observed, the present findings support further investigation of Hist 5 as a peptide scaffold for mechanistic studies and future optimization rather than as a direct therapeutic candidate in its native form.

The direct activity of Hist 5 against *T. cruzi* epimastigotes aligns with previous reports highlighting AMPs as promising alternatives against kinetoplastid parasites [22]. Other structural families, such as apidaecin, magainin II, melittin, and cecropin A, have previously shown robust in vitro anti-trypanosomal activity [19,20,21]. Notably, we observed that the IC_50_ values of Hist 5 increased substantially at 72 h (135.23 ± 0.56 μg/mL) and 96 h (156.21 ± 0.32 μg/mL) compared to the lowest value achieved at 48 h (61.5 ± 0.5 μg/mL), indicating attenuation of its inhibitory activity during prolonged incubation. Consistent with this observation, the washout assay showed that epimastigotes previously exposed to 61.5, 106, or 212 μg/mL Hist 5 resumed proliferation after peptide withdrawal. Although parasite densities remained below those of untreated controls, recovery was observed at all three concentrations, whereas benznidazole-treated parasites showed a more sustained suppression of proliferation after drug removal. These findings indicate that the effect of Hist 5 is at least partially reversible and suggest that continuous peptide exposure may be required to maintain the maximal inhibition of parasite growth.

One possible explanation for the reduced activity observed during prolonged incubation is limited peptide stability under the experimental conditions. Hist 5 is a linear, histidine-rich peptide, so it is highly susceptible to proteolysis [23]. In addition, *T. cruzi* expresses abundant proteolytic enzymes into the medium, such as the cysteine protease cruzain (cruzipain) [24,25], which could potentially contribute to peptide degradation. However, the stability of Hist 5 in the parasite culture system was not directly evaluated in the present study; therefore, peptide degradation remains a hypothesis that requires experimental confirmation. Future studies evaluating Hist 5 stability in culture medium and in the presence of parasites will be necessary to clarify this possibility. Strategies such as cyclization or the incorporation of D-amino acids could subsequently be explored to improve peptide stability [26,27]. The activity observed against *T. cruzi* epimastigotes (IC_50_ = 61.5 µg/mL, approximately 20 µM at 48 h) also suggests that this parasite stage may be less susceptible to Hist 5 than some other microorganisms previously evaluated. In *Leishmania*, Hist 5 has been reported to exhibit LC_50_ values of 7.3 µM for promastigotes and 14.4 µM for amastigotes [17], whereas activity against *Candida* spp. has been reported at micromolar concentrations, including IC_50_ values of approximately 3 µM under specific assay conditions and MIC_50_ values of 10–20 µg/mL against several non-*Candida albicans* species [28]. The comparatively higher concentration required to affect *T. cruzi* epimastigotes may reflect differences in membrane composition, peptide uptake, or cellular bioenergetics. Nevertheless, these values should not be interpreted as directly equivalent because the studies differ in target organism, developmental stage, assay conditions, exposure time, and experimental endpoint.

Importantly, the antiparasitic effect of Hist 5 was observed under conditions in which Vero cells and RAW264.7 macrophages showed only partial reductions in viability. This point is relevant because host-cell toxicity remains one of the major limitations in the development of new antiparasitic compounds [29,30]. However, these findings should be interpreted cautiously. Because 50% cytotoxicity was not reached in either Vero cells or RAW264.7 macrophages within the tested concentration range, formal CC_50_ values and selectivity indices could not be calculated in the present study. Therefore, the cytotoxicity results should not be considered definitive evidence of parasite selectivity. Future studies using broader concentration ranges, infected host-cell models, and clinically relevant *T. cruzi* stages will be necessary to better define the selectivity profile and therapeutic window of Hist 5.

Beyond reducing viability, Hist 5 induced profound ultrastructural collapse in the treated epimastigotes. TEM revealed early mitochondrial swelling and distension of the kinetoplast region at 48 h, which progressed to severe vacuolization and complete disorganization of endomembrane systems by 72 h. These structural defects correlate closely with the observed induction of apoptosis-like features. The significant increase in TUNEL-positive parasites (26.1%) indicates DNA strand breaks, while Annexin V-FITC labeling confirmed phosphatidylserine exposure on the outer leaflet of the plasma membrane in a distinct subpopulation (10.3%). The difference between the percentages obtained by TUNEL and Annexin V-FITC staining may be explained by the fact that these assays detect distinct cellular events and may reflect different stages or manifestations of apoptosis-like cell death. Annexin V-FITC identifies phosphatidylserine externalization at the plasma membrane, whereas TUNEL detects DNA strand breaks. In protozoan parasites, including trypanosomatids, apoptosis-like phenotypes are usually defined by the combined detection of several markers, such as mitochondrial dysfunction, phosphatidylserine exposure, chromatin condensation, and DNA fragmentation, rather than by a single assay [31,32,33]. Therefore, the higher proportion of TUNEL-positive parasites compared with Annexin V-positive parasites suggests that at the evaluated time point, Hist 5-induced damage was more prominently associated with DNA fragmentation than with detectable phosphatidylserine exposure. These results should be interpreted as complementary evidence of apoptosis-like alterations rather than as equivalent measurements of the same event.

The partial recovery observed after Hist 5 withdrawal suggests heterogeneity in the response of the parasite population. Although proliferation resumed after peptide removal, parasite densities remained below those of the washed control, indicating that not all parasites recovered their proliferative capacity to the same extent. This population-level response, together with the apoptosis-like alterations detected in a fraction of Hist 5-treated parasites, supports the interpretation that susceptibility to the peptide is not uniform across the parasite population. In *Leishmania* spp., Hist 5 has been reported to undergo intracellular translocation and accumulate in the mitochondrion, where it interferes with ATP synthesis and mitochondrial bioenergetics [17]. This previously described mitochondrial activity is consistent with the mitochondrial and kinetoplast alterations observed in *T. cruzi* epimastigotes in the present study. However, the intracellular localization of Hist 5 and its effects on mitochondrial bioenergetics were not directly evaluated here; therefore, a similar mechanism in *T. cruzi* remains to be experimentally demonstrated. Our TEM findings are therefore consistent with mitochondrial and kinetoplast involvement in the cellular response of *T. cruzi* to Hist 5. However, the temporal relationship between mitochondrial damage and the other apoptosis-like alterations observed in this study cannot be established from ultrastructural analysis alone. Moreover, programmed cell death pathways in protozoan parasites differ substantially from canonical mammalian apoptosis [34]. Further studies evaluating mitochondrial membrane potential (ΔΨm), metacaspase-like activity, intracellular calcium homeostasis, and peptide intracellular localization would help clarify the sequence of cellular events associated with Hist 5 exposure. Hist 5 exposure was also associated with modulation of the parasite redox and nitrosative status. Unlike benznidazole, whose antiparasitic activity involves prodrug activation by trypanosomal nitroreductases and the generation of reactive intermediates [35], Hist 5 induced a significant but moderate increase in ROS levels alongside a prominent elevation of nitrite/NO-associated responses after 48 h. This combined response is potentially relevant because ROS functions in *T. cruzi* are complex and context-dependent. Whereas excessive ROS accumulation can promote oxidative damage [36], moderate ROS levels can paradoxically act as signaling molecules that stimulate parasite proliferation [37]. The concurrent increase in ROS and nitrite/NO-associated responses induced by Hist 5 could potentially favor the formation of reactive nitrogen species such as peroxynitrite (ONOO−). Peroxynitrite is a highly reactive and destructive nitrogen species capable of lipid peroxidation, protein nitration, and direct DNA damage [38], which would explain the focal plasma membrane discontinuities and nuclear fragmentation captured in our TEM and TUNEL assays. However, peroxynitrite formation was not directly measured in this study, and its involvement should consequently be considered a mechanistic hypothesis. Overall, the antiparasitic effect of Hist 5 may involve the disruption of redox and nitrosative homeostasis through pathways that differ from those associated with conventional nitroheterocyclic drugs.

An important limitation of the present study is that all experiments were performed using epimastigotes, a replicative stage of *T. cruzi* that develops in the triatomine vector and is not the form responsible for establishing mammalian infection. Epimastigotes were selected because they provide a reproducible experimental model for the mechanistic analyses performed here; however, susceptibility to Hist 5 cannot be assumed to be equivalent across developmental stages. In particular, the activity of Hist 5 against infective trypomastigotes and intracellular amastigotes remains to be determined. Studies using these clinically relevant stages and infected host-cell models will therefore be essential to establish the biological and translational relevance of the present findings.

In conclusion, our results demonstrate that Hist 5 exerts antiparasitic activity against *T. cruzi* epimastigotes and induces multiple cellular alterations involving mitochondrial–kinetoplast integrity, redox and nitrosative homeostasis, and apoptosis-like features. The partial recovery of parasite proliferation after Hist 5 withdrawal indicates a trypanostatic component under the experimental conditions evaluated, whereas the persistent reduction in parasite density and the cellular alterations observed suggest that a fraction of the parasite population undergoes more severe damage. Together with the relatively high concentrations required for activity, the attenuation of the inhibitory effect during prolonged incubation, and the limited effects observed in mammalian cells, these findings identify important limitations of the native peptide while supporting its further investigation as a molecular scaffold for mechanistic studies and future optimization. Given the limitations of current Chagas disease chemotherapy, including toxicity, adverse effects, and reduced efficacy during the chronic phase [39,40], peptide-based molecules such as Hist 5 may remain valuable tools for identifying novel parasite vulnerabilities and guiding the development of optimized antiparasitic strategies.

## 4. Materials and Methods

### 4.1. Parasites

Epimastigotes of the *T. cruzi* Queretaro strain (TBAR/MX/0000/Queretaro) were cultured at 28 °C in LIT medium (liver infusion-tryptose: 0.5% tryptose, 0.5% liver infusion, 0.2% glucose, 0.4% NaCl, 0.04% KCl, 0.42% Na_2_HPO_4_) supplemented with 10% fetal bovine serum (FBS) (Biowest; Nuaillie, France); antibiotic mixture, 100 U penicillin/mL and 100 µg streptomycin/mL (Sigma-Aldrich, Darmstadt, Germany), and 25 µg/mL hemin (Santa Cruz; Dallas, TX, USA) [41].

### 4.2. Hist 5

Lyophilized human Hist 5 was purchased from Sigma-Aldrich (St. Louis, MO, USA; Cat. No. H6027), with the following amino acid sequence: Asp-Ser-His-Ala-Lys-Arg-His-His-Gly-Tyr-Lys-Arg-Lys-Phe-His-Glu-Lys-His-His-Ser-His-Arg-Gly-Tyr. The peptide was reconstituted in 1 mL of 10 mM phosphate-buffered saline (PBS; pH 7.0) containing 0.9% (*w*/*v*) NaCl.

### 4.3. Antitrypanosomal Activity Assays Against T. cruzi Epimastigotes

*T. cruzi* epimastigotes (5 × 10^5^ parasites/mL) were obtained from logarithmic-phase cultures between days 5 and 8. Parasites were harvested and washed three times with LIT medium by centrifugation at 750× *g* for 20 min. The parasite suspension was adjusted to 5 × 10^5^ parasites/mL and seeded in 96-well microplates. Epimastigotes were exposed to different concentrations of Hist 5 (26, 53, 106, 212, and 424 µg/mL) and incubated at 28 °C for 5 days. Benznidazole (19.34 µg/mL) was included as the reference drug, whereas untreated epimastigotes were used as the negative control. At each evaluated time point, parasite growth and viability were assessed by light microscopy using a Neubauer chamber. Viable parasites were identified based on motility and trypan blue dye exclusion, whereas trypan blue-positive parasites were considered non-viable. Viable parasite density was quantified and used to calculate the concentration required to reduce the number of viable epimastigotes by 50% relative to the untreated controls.

To evaluate whether the inhibitory effect of Hist 5 persisted after peptide withdrawal, a washout assay was performed after 48 h of exposure. This time point was selected because it corresponded to the reference IC_50_ determination and to the conditions used for most subsequent assays. Epimastigotes were exposed to Hist 5 at the 48-h IC_50_ (61.5 µg/mL) and at 106 and 212 µg/mL. Benznidazole (19.34 µg/mL) and untreated parasites were included as reference and growth controls, respectively. After 48 h of exposure, parasite suspensions were centrifuged at 750× *g* for 10 min, the supernatants were discarded, and the parasites were washed twice with phosphate-buffered saline (PBS, pH 7.2). Parasites were then resuspended in fresh LIT medium without Hist 5 or benznidazole and maintained at 28 °C. Parasite growth and viability were evaluated at 24, 48, 72, 96, and 120 h after treatment withdrawal by Neubauer chamber counting and trypan blue exclusion, as described above.

### 4.4. Cytotoxicity Assays on Mammalian Cells

The cytotoxic effect of Hist 5 was evaluated on Vero cells (ATCC CCL-81) and murine RAW264.7 macrophages (ATCC TIB-71) by measuring resazurin reduction to resorufin (PrestoBlue, Invitrogen by ThermoFisher Scientific, Waltham, MA, USA). Cells were seeded in 96-well plates (Costar, Corning Life Sciences, Corning, NY, USA) at a density of 1 × 10^5^ cells/well in D-MEM (Gibco, Grand Island, NY, USA) or RPMI-1640 medium (Gibco, Grand Island, NY, USA), and incubated for 12 h at 37 °C under a 5% CO_2_ atmosphere. Hist 5 was added at different concentrations (5.6, 11.3, 22.67, 45.34, 61.5, 90.68 µg/mL) and incubated for 24, 48, and 72 h. After incubation, cells were washed with HBSS, and 10 µL of PrestoBlue reagent mixed with 90 µL of Hank’s solution was added. Plates were incubated for 3 h at 37 °C with 5% CO_2_, and the absorbance intensity was measured at a wavelength of 570 nm using a Thermo Labsystems 354 Multiskan Ascent Microplate Reader (Thermo Fisher Scientific, Waltham, MA, USA). Cells treated with benznidazole (19.34 µg/mL) served as the positive control, whereas cells cultured exclusively in their respective media served as negative controls. Readings were obtained using Ascent software version 2.6 for multiple layers.

### 4.5. Determination of Half-Maximal Inhibitory Concentration (IC_50_)

The half-maximal inhibitory concentration (IC_50_) for both Hist 5 and benznidazole against *T. cruzi* epimastigotes was determined from the dose–response curves (Section 4.3). The IC_50_ values were computed by fitting the experimental dose–response data to a non-linear regression model (log[inhibitor] vs. normalized response with a variable slope) using GraphPad Prism software (version 8.0, GraphPad Software, San Diego, CA, USA).

### 4.6. Transmission Electron Microscopy (TEM)

For ultrastructural analysis, a suspension of epimastigotes (5 × 10^6^ cells) was incubated with Hist 5 for 48 and 72 h. After treatment, parasites were washed three times with fresh PBS (pH 5.2) at 4 °C, rinsed with 0.15 M cacodylate buffer, and fixed in Karnovsky’s solution for 1 h at room temperature. The samples were then transferred to 0.1 M cacodylate buffer, post-fixed in 1% (*w*/*v*) osmium tetroxide, dehydrated through a graded ethanol series (30%, 50%, 70%, 90%, and 100%) followed by propylene oxide (1 h), and embedded in Poly/Bed 812/DMP30 (Polysciences, Warrington, PA, USA). Ultrathin sections were examined and photographed using a JEOL JEM-1200 EXII transmission electron microscope (JEOL, Tokyo, Japan) [42].

### 4.7. TUNEL Assay for Chromatin Fragmentation

DNA strand breaks associated with apoptosis-like cell death were evaluated using the terminal deoxynucleotidyl transferase dUTP nick end labeling (TUNEL) assay (In Situ Cell Death Detection Kit; Roche Diagnostics, Mannheim, Germany). Epimastigotes previously treated with Hist 5 (61.5 µg/mL) were washed twice with PBS containing 1% (*w*/*v*) bovine serum albumin (BSA) at 4 °C, fixed in a freshly prepared 4% (*w*/*v*) paraformaldehyde solution in PBS (pH 7.4) for 30 min at room temperature, and centrifuged at 300× *g* for 10 min.

The parasites were then permeabilized with a solution of 0.1% (*v*/*v*) Triton X-100 in 0.1% (*w*/*v*) sodium citrate for 2 min on ice (4 °C), washed twice with PBS (pH 7.4), and resuspended in 50 μL of the TUNEL reaction mixture for 60 min at 37 °C in a humidified dark chamber. After two subsequent washes with PBS, samples were analyzed by flow cytometry. As a negative control, fixed and permeabilized epimastigotes were incubated in the label solution without the terminal deoxynucleotidyl transferase enzyme. Epimastigotes treated with benznidazole (19.34 µg/mL) served as the positive control for DNA fragmentation. Fluorescence was measured using a FACSCalibur flow cytometer (Becton Dickinson, San Diego, CA, USA). Fluorescence compensation was established using parasites incubated solely with the label solution. Samples were analyzed in quintuplicate, acquiring 10,000 events per sample.

### 4.8. Annexin-V/PI Binding Assay

Phosphatidylserine (PS) externalization on the surface of *T. cruzi* epimastigotes was analyzed using the Annexin-V-FLUOS Staining Kit (Roche Diagnostics). Epimastigotes treated with Hist 5 (61.5 µg/mL) were washed twice with PBS (pH 7.4) and resuspended in 100 µL of staining solution (Annexin-V-Fluos in HEPES buffer [10 mM HEPES/NaOH, pH 7.4, 140 mM NaCl, 5 mM CaCl_2_], supplemented with 50 µg/mL propidium iodide [PI]) for 15 min at 25 °C in the dark. Epimastigotes treated with 4 µg/mL camptothecin (Roche) for 3 h were used as a positive control. Samples were acquired on a FACSort flow cytometer equipped with CellQuest software (version 5.2.1, BD Biosciences, San Jose, CA, USA). Data analysis was performed using FlowJo software (version 10, Becton Dickinson, Milpitas, CA, USA). Results are expressed as percentages of viable (AnnV−/PI−), early apoptotic (AnnV+/PI−), or non-viable (AnnV+/PI+ or AnnV−/PI+) cells.

### 4.9. Nitric Oxide (NO) Production

Nitric oxide production by *T. cruzi* epimastigotes was evaluated by measuring nitrite accumulation in the culture supernatants using the Griess Reagent System (Life Technologies, Carlsbad, CA, USA). Parasites (1 × 10^6^ cells/mL) were stimulated with 61.5 µg/mL Hist 5 for 48 h. Supernatants were collected by centrifugation, and 50 µL of each sample was transferred to a 96-well plate and mixed with 20 µL of Griess reagent (1% sulfanilamide in 5% phosphoric acid and 0.1% N-1-naphthylethylenediamine dihydrochloride; Sigma-Aldrich). After incubation for 30 min at room temperature, absorbance was recorded at 550 nm using a Multiskan SkyHigh microplate reader (Thermo Scientific, Singapore). Unconditioned culture medium was used as a blank. Nitrite concentrations were calculated using a sodium nitrite (NaNO_2_) standard curve. Untreated epimastigotes served as the baseline control.

### 4.10. Production of Reactive Oxygen Species (ROS)

Endogenous ROS generation in epimastigotes was measured using the cell-permeable probe 2′,7′-dichlorodihydrofluorescein diacetate (H_2_ DCFDA; Sigma-Aldrich). Parasites stimulated with Hist 5 (61.5 µg/mL) were incubated in 96-well plates for 24 h. Cells were then loaded with 100 µg/mL H_2_ DCFDA for 30 min at room temperature in the dark.

Following incubation, parasites were washed twice with PBS (pH 7.2), detached using a 0.02% (*w*/*v*) EDTA solution, and resuspended in PBS (pH 7.2) supplemented with 1% (*v*/*v*) FBS. Samples were immediately analyzed on a FACSCanto II flow cytometer using FlowJo v10 software (Becton Dickinson). Untreated epimastigotes were used as a negative control.

### 4.11. Statistical Analysis

Data obtained from antimicrobial activity, cell viability, ROS production, nitrite levels, and cell death assays (TUNEL and Annexin V) were expressed as the mean ± standard deviation (SD) from five independent experiments (*n* = 5).

For experiments evaluating variables across multiple groups and time points, statistical significance was determined using a two-way analysis of variance (ANOVA), followed by Tukey’s post hoc test for multiple comparisons. Non-linear regression analysis (four-parameter logistic curve) was utilized to calculate the half-maximal inhibitory concentration (IC_50_). A *p*-value < 0.05 was considered statistically significant (* *p* < 0.05, ** *p* < 0.01, *** *p* < 0.001). All statistical analyses and graphical representations were performed using GraphPad Prism software (version 8.0, GraphPad Software, San Diego, CA, USA).

## 5. Conclusions

This study provides the first evidence that the human salivary peptide Hist 5 affects *T. cruzi* epimastigotes in vitro. Hist 5 exposure reduced parasite viability/proliferation and induced multiple cellular alterations, including ultrastructural damage, membrane alterations, cytoplasmic vacuolization, organelle disruption, phosphatidylserine externalization, DNA fragmentation, and changes in oxidative/nitrosative responses. These findings suggest that Hist 5 alters parasite homeostasis through multiple damage-associated processes.

However, the activity of Hist 5 was observed at relatively high concentrations, the inhibitory effect decreased after prolonged incubation, and partial reductions in mammalian cell viability/metabolic activity were observed under some experimental conditions. Therefore, Hist 5 should not yet be considered a direct therapeutic candidate in its current form. Rather, our results support Hist 5 as a useful peptide scaffold for studying *T. cruzi* susceptibility to antimicrobial peptides and for guiding future optimization strategies. Further studies are required to evaluate peptide stability, reversibility of the antiparasitic effect, formal selectivity indices, activity against trypomastigotes and intracellular amastigotes, and efficacy in more complex infection models.

## Figures and Tables

**Table 1:** IC_50_ values of Hist 5 and benznidazole against *T. cruzi* epimastigotes at different incubation times. Values are expressed as mean ± standard deviation. IC_50_ values were calculated from dose–response curves using nonlinear regression analysis.

Treatment	Time (h)	IC_50_ (μg/mL)
Benznidazole	24	9.45 ± 0.12
Benznidazole	48	19.34 ± 0.5
Benznidazole	72	15.34 ± 0.23
Benznidazole	96	6.57 ± 0.54
Hist 5	24	98.89 ± 0.24
Hist 5	48	61.5 ± 0.5
Hist 5	72	135.23 ± 0.56
Hist 5	96	156.21 ± 0.32

## Data Availability

The original contributions presented in this study are included in the article. Further inquiries can be directed to the corresponding author.

## Abbreviations

The following abbreviations are used in this manuscript:

DNA	Deoxyribonucleic acid
H_2_DCFDA	2′,7′-dichlorodihydrofluorescein diacetate
ROS	Reactive oxygen species
NO	Nitric oxide
AMPs	Antimicrobial peptides
ATP	Adenosine triphosphate
IC_50_	Half maximal inhibitory concentration
TEM	Transmission electron microscopy
PMA	Phorbol 12-myristate 13-acetate
FITC	Fluorescein isothiocyanate
TUNEL	Terminal deoxynucleotidyl transferase dUTP nick-end labeling
NADH	Nicotinamide adenine dinucleotide
